# Alpha Thalassemia/Intellectual Disability X-Linked Deficiency Sensitizes Non-Small Cell Lung Cancer to Immune Checkpoint Inhibitors

**DOI:** 10.3389/fonc.2020.608300

**Published:** 2020-12-21

**Authors:** Tao Hou, Shun Jiang, Yapeng Wang, Yangchun Xie, Haixia Zhang, Yeqian Feng, Fang Ma, Jin’an Ma, Xianling Liu, Chunhong Hu

**Affiliations:** Department of Oncology, The Second Xiangya Hospital, Central South University, Changsha, China

**Keywords:** lung cancer, immune checkpoint inhibitor, CRISPR, tumor supperssor gene, α- thalassemia/intellectual disability syndrome x-linked

## Abstract

**Background:**

The immune checkpoint inhibitors (ICIs) have achieved great success in the treatment of non-small cell lung cancer (NSCLC) patients. However, the response rate is low. The molecular mechanism involved in the effectiveness of ICIs remains to be elucidated.

**Methods:**

ATRX mutation incidence among human cancers was analyzed from TCGA database. Atrx-deficient Lewis lung cancer cell line (LLC-sgAtrx) was established *via* AAV-CRISPR. Subcutaneous and metastasis models were established by subcutaneous and intravenous injection of LLC-sgAtrx and LLC-sgNTC cells into female C57BL/6 mice. The mice were treated with anti-PD1, anti-CLTA4 or Rat IgG2a. Tumor volume was determined by Vernier calipers and the IVIS imaging system. The proportions of CD3+ T cells, CD45+ immune cells, and the expression of pMHC I and PDL1 were determined by flow cytometry. The T cell cytotoxicity was determined by co-culture experiment.

**Results:**

TCGA data showed that Atrx is a tumor suppressor mutated at high frequency among various human cancers. The tumor volume of mice bearing LLC-sgAtrx was significantly shrinked and the median survival of mice was significantly longer after anti-PD1 and anti-CTLA4 treatment. Flowcytometry results showed that Atrx deficiency increase the penetration of CD3+ T cell into the tumor microenvironment and enhanced antigen presentation after IFNγ stimulation. Additionally, the tumor cells with Atrx deficiency were more easily to be damaged by T cells under IFNγ stimulation.

**Conclusion:**

The present study demonstrated that Atrx deficiency sensitize lung cancer cells to ICIs by multiple mechanisms. And ATRX may serve as a promising biomarker for ICIs which helps patient stratification and decision making.

## Introduction

Lung cancer is the leading cause of death worldwide, among which non-small cell lung cancer (NSCLC) comprises more than 80% of cases (1). During the past decade, immune therapy has been shown to be the cornerstone of NSCLC treatment (2–4). However, most patients do not respond to immune checkpoint inhibitors (ICIs), with a general response rate of approximately 23% in previously treated patients and 41% in treatment-naïve patients (5). The biomarkers that could be used to predict the effectiveness of ICIs is still not well defined. PD-L1 expression is the most well studied biomarker, and it is reported that patients with PD-L1 expression on at least 50% of tumor cells response well to pembrolizumab, with an objective response rate (ORR) of 44.8%, and the median progression free survival was 10.3 months, which significantly higher than chemotherapy (6). Besides, tumor mutation burden (TMB) is another useful biomarker, and patients with TMB≥10/Mb have higher ORR to anti–PD-1+ anti-CTLA4 antibodies compared with chemotherapy (7).Thus, the Food and Drug Administration (FDA) has approved the use of pembrolizumab and nivolumab+ipilimumab in the first-line treatment of NSCLC patients with PD-L1 expression or high TMB However, the stratification of patients still needs further presicion. And factors that affect ICI effectiveness still need to be further elucidated. Therefore, exploring effective biomarkers to facilitate patient stratification is the major challenge for precision ICI treatment.

Alpha thalassemia/intellectual disability X-linked (ATRX) is a protein containing an ATPase/helicase domain; thus, it belongs to the switch/sucrose nonfermentable (SWI/SNF) family of chromatin remodeling proteins. It is found to undergo cell cycle-dependent phosphorylation, which regulates its nuclear matrix and chromatin association and suggests its involvement in gene regulation at interphase and chromosomal segregation in mitosis. Atrx has been found frequently mutated in glioma and pancreatic neuroendocrine tumors and is an independent prognosis biomarker (8, 9) and promising therapeutic target (10). However, the role of Atrx mutation in NSCLC immune therapy has not been reported. In the present study, we demonstrated that Atrx deficiencies sensitize nonsmall cell lung cancer cells to ICI therapies in a syngeneic mice model.

## Materials and Methods

### Cell Line

The Lewis lung cancer cell line was purchased from the Cell Bank of the Chinese Academy of Sciences and was cultured at 37°C in 5% CO_2_ in Dulbecco’s modified Eagle’s medium (DMEM) supplemented with 10% fetal bovine serum (FBS) (Gibco) and antibiotics (100 units/ml penicillin and 100 μg/ml streptomycin).

### Lentiviral Cas9 Preparation and Infection

sgRNAs targeting Atrx (sgAtrx) or targeting nowhere in the mouse genome (sgNTC) were cloned into lentiCRISPR-v2 (Addgene). HEK293T cells were transfected with lentiCRISPR-v2 and the packaging plasmids psPAX and pMD2.G (both Addgene) using polyethylenimine. After 24 h, medium was replaced by DMEM (Gibco) containing 10% fetal bovine serum. Twenty-four hours later, lentivirus-containing supernatant was harvested, filtered and stored at −80°C. Virus was titrated by infecting LLC cells at a number of different concentrations, followed by the addition of 2 mg/ml puromycin at 24 h postinfection to select the transduced cells. For lentiviral transduction, 6 × 10^5^ LLC cells were seeded per well in a 6-well plate (Corning), and lentivirus was added in combination with 8 µg/ml polybrene (Sigma-Aldrich). After 24 h, cells were selected with 2 mg/ml puromycin for at least 7 days. The T7E1 assay was used to determine the knockout efficacy of Atrx.

### Establishment of the Lewis Lung Cancer Mouse Models

All experiments were performed according to the institutional guidelines for the care and use of animals and were approved by the Animal Care and Use Committee of the Second Xaingya Hospital, Central South University. To establish LLC mouse models with subcutaneous tumors and metastatic tumors, LLC-sgNTC and LLC-sgAtrx cells were suspended in 150 μl of PBS at a density of 1 × 10^6^/ml. Then, these cell suspensions were subcutaneously injected or intravenously injected *via* the tail vein into C57BL/6 mice with a 1-ml syringe. The anti-CTLA4 and anti–PD-1 were given at a dose of 200 ug/mice at 9, 12, and 15 days after the establishment of models. The sizes of the subcutaneous tumors were measured by Vernier calipers every 3 days [tumor volume = 1/2 × (L × W)^2^]. For the metastasis model, tumor volume was monitored *in vivo* by bioluminescence detected by the IVIS imaging system (Bruker, USA) once every month. A D-luciferin potassium salt solution (Goldbio. St. Louis, MO, USA) was injected intraperitoneally (150 mg/kg), and 10–15 min after injection, the mice were imaged for *in vivo* tumor growth using an IVIS machine (PerkinElmer). Living Image Software (Bruker MI, USA) was used to measure the total flux of the metastatic lung tumor.

### Flow Cytometry

Single-cell suspensions of tumors were prepared using a gentle MACS tissue dissociation system. The purified cells were stained as follows: Panel 1: anti-CD45-PE, anti-CD3-APC; or Panel 2: anti-SIINFEKL-H2K^b^-APC/Cy7 and anti-PD-L1-APC/Cy7. Antibody incubations were performed on ice, with the cells being fixed in 1% paraformaldehyde and analyzed on a BD LSRFortessa (BD Bioscience). All flow antibodies were used at 1:100 dilutions for staining. For surface staining, cells were blocked with anti-Fc receptor anti-CD16/CD32 and then stained with surface marker antibodies in staining buffer consisting of 2% FBS in PBS on ice for 30 min. Samples were washed twice with 2% FBS in PBS before analysis.

### In Vitro Antigen Presentation and Cytotoxicity Assays

To test the effect of IFNγ on surface peptide-MHCI presentation, 2 × 10^5^ LLC-sgAtrx or LLC-sgNTC cells were seeded per well in 12-well culture plates (Corning). Then, 10 ng/ml IFNγ was added, and cells were incubated for 24–48 h. The treated cells were collected and washed twice with 2% FBS in PBS. Then, the cells were stained with SIINFEKL-H-2K^b^-APC/Cy7 or PDL1-APC/Cy7 for 30 min on ice and washed twice with 2% FBS in PBS before flow cytometry analysis. For *in vitro* cytotoxicity assay, 2 × 10^4^ LLC-sgAtrx or LLC-sgNTC cells were seeded per well in a 96-well white polystyrene plate (Corning). CD8 T cells were admixed in serial dilutions (0, 1:2, 1:1 ratio), and 10 ng/ml IFNγ was added. After 24 h, cancer cell killing was measured by adding 150 μg/ml D-luciferin (ThermoFisher) using a multichannel pipette. Luciferase intensity was measured with a plate reader (Multiscan FC Microplate Reader, Thermo Fisher).

### Analysis of Atrx Mutation Status in Patient Cohorts

To determine the Atrx mutation status in clinical patient data, the cBioPortal was queried across the PanCancer TCGA cohorts. The OQL specifiers “MUT HOMDEL” were used for all mutation and deletion analyses. Statistical significance was assessed by the two-tailed Mann-Whitney test.

### Statistical Analysis

The unpaired two-tailed Student t-test and one-way analysis of variance (ANOVA) were used for intergroup comparisons. The Kaplan-Mayer method was used for survival analysis. All statistical analyses were conducted using SPSS (version 22.0) and GraphPad (Version 7.0). All data are presented as the mean ± SD (standard deviation), and P values < 0.05 were considered statistically significant.

## Results

### Alpha Thalassemia/Intellectual Disability X-Linked Is Highly Mutated in Multiple Human Cancer Types

**Figure 1:**
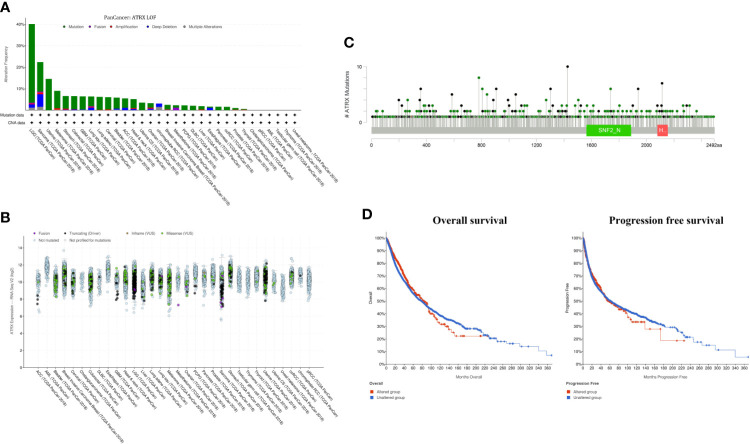
Atrx alteration is prevalent among human cancers. **(A)** percentage of Atrx alteration among common human cancers. **(B)** Expression of different Atrx mutation forms among common human cancers. **(C)** the mutation site within Atrx domains. **(D)** Differences in overall survival and progression free survival between Atrx altered and unaltered patients.

**Figure 2:**
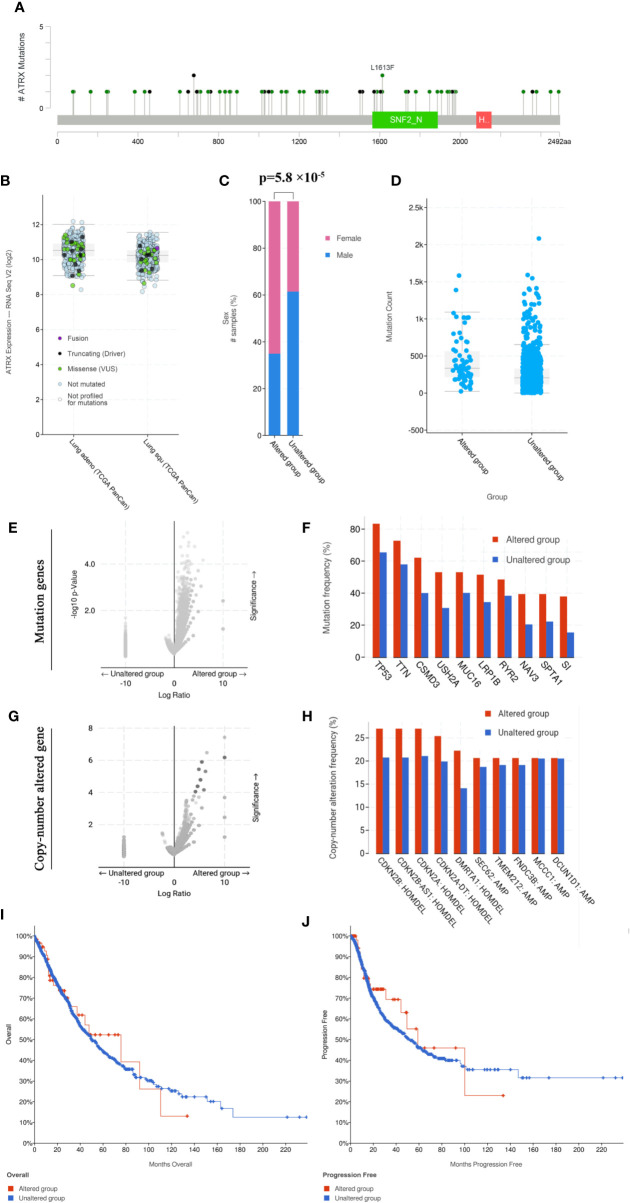
Atrx mutation characteristics in NSCLC patients. **(A)** the mutation site of Atrx in NSCLC patients. **(B)** Different Atrx mutations forms in adenocarcinoma and squamous cell carcinoma. **(C)** Gender differences in patients with Atrx mutation. **(D)** The association between Atrx mutation and total mutation burden in NSCLC. **(E)** Comparison of tumor mutation genes between Atrx altered and unaltered patient groups. **(F)** the most frequently mutated gene accompanied with Atrx mutation. **(G)** Comparison of tumor copy-number altered genes between Atrx altered and unaltered patient groups. **(H)** the most frequent gene with altered copy number accompanied with Atrx mutation. **(I)** Differences in overall survival between Atrx altered and unaltered patients. **(J)** Differences in progression free survival between Atrx altered and unaltered patients.

Cross-cancer analysis of the TCGA database shows that low-grade glioma has the highest incidence of Atrx mutation rate of approximately 40%, followed by sarcoma and uterine cancer (**Figure 1A**). The mutation rate of Atrx in lung cancer is approximately 8%. The most common mutation forms in lung cancer are truncation and missense (**Figure 1B**). The most common mutation site is the SNF2_N domain (**Figure 1C**). The presence of Atrx alteration has no significant effect on overall survival and progression-free survival in a cross-cancer population (**Figure 1D**). As in NSCLC patients, the most common mutation site is also in the SNF2_N domain (**Figure 2A**). The most common mutation types in both adenocarcinoma and squamous cell carcinoma are truncation and missense (**Figure 2B**). Atrx mutation in lung cancer patients has a gender difference (**Figure 2C**), and patients with Atrx mutation have a higher mutation burden than those without Atrx mutation, though the difference is not significant (**Figure 2D**). Atrx mutation is always accompanied by higher levels of gene mutations, including TP53, TTN, CSMD3, and USH2A (**Figures 2E, F**), and gene copy number variations, including CDKN2B, CDKN2A, and DMRTA1 (**Figures 2G, H**). NSCLC patients with Atrx mutation have slightly longer median overall survival and progression-free survival, but the difference is not statistically significant (**Figures 2I, J**). Given the prevalence of Atrx mutations in NSCLC patients, we sought to investigate whether Atrx deficiency affects ICI responsiveness in lung cancer models.

### Alpha Thalassemia/Intellectual Disability X-Linked Deficiency Sensitizes Nonsmall Cell Lung Cancer to Immune Checkpoint Inhibitors

**Figure 3:**
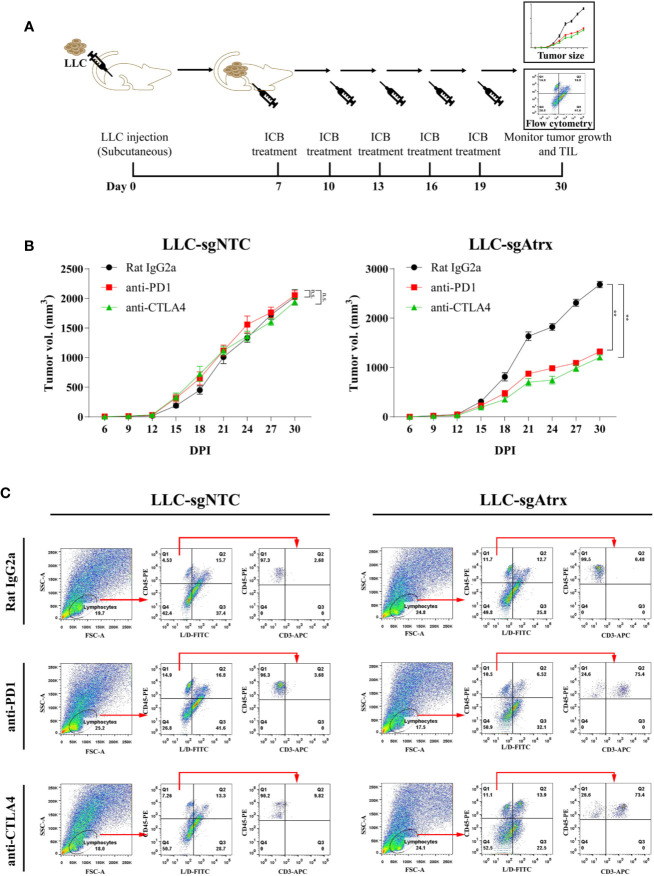
Atrx deficiency sensitizes NSCLC to ICI treatment in subcutaneous syngeneic mouse model. **(A)** Experimental design for subcutaneous tumor model establishment and analysis of tumor-infiltrating immune cells. **(B)** Tumor volume curves of mice bearing LLC tumors with *and without Atrx* knockout. Neither aCTLA4 (n = 5) nor aPD1 (n = 5) treated mice showed a significant volume difference in Atrx-expression mice, compared with control group (n = 5) (*P* = 0.8712, 0.8981). Both aCTLA4 (n = 5) and aPD1 (n = 5) treated mice showed a significant volume difference in Atrx-deficient mice, compared with control group (n = 5) (*P* = 0.005, 0.008). **(C)** T cell proportion in the microenvironment of LLC generated tumors with and without Atrx deficiency after ICI or isotype antibody treatment. Quantification of CD45+ immune cells in tumor sections from Atrx expression, or Atrx-deficient tumors, with or without anti-PD1, anti-CTLA4 treatment. Two-tailed unpaired t-test, CD45+ cells in LLC-sgNTC group: anti-PD1 (n = 10) vs. control (n = 10), P = 0.8713; anti-CTLA4 (n = 10) vs. control (n = 10), P = 0.1054. CD45+ cells in LLC-sgAtrx group: anti-PD1 (n = 10) vs. control (n = 10), P = 0.0004; anti-CTLA4 (n = 10) vs. control (n = 10), P = 0.0005. **P<0.01. n.s., not significant.

**Figure 4:**
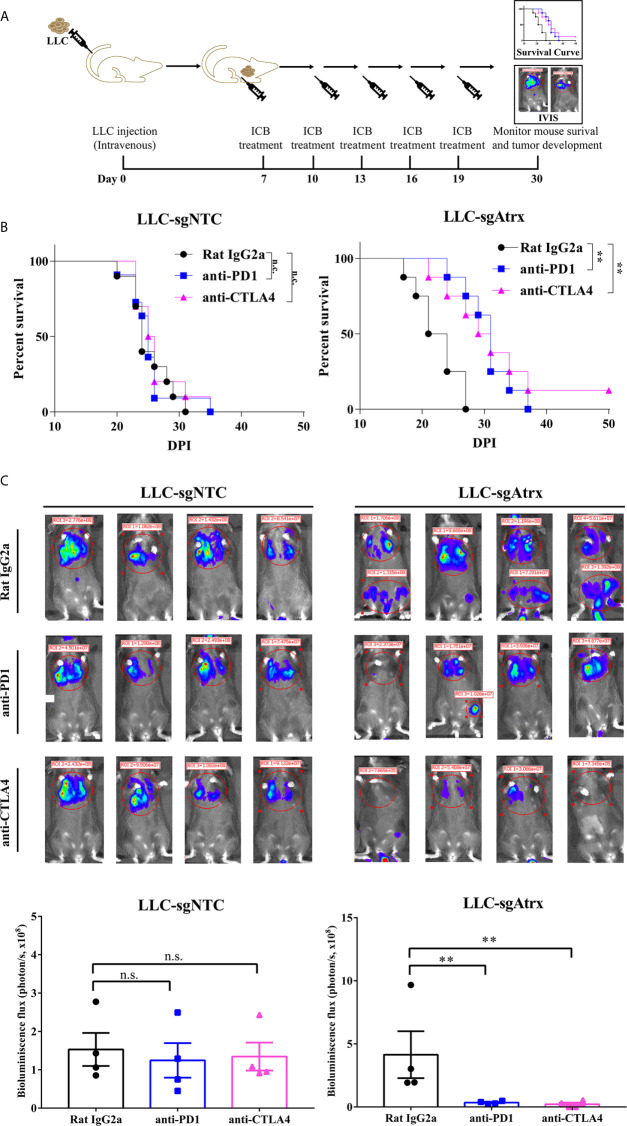
Atrx deficiency sensitizes NSCLC to ICI treatment in orthotopic mouse model. **(A)** experimental design for establishment of the orthotopic mouse model by intravenous seeding of tumor cells to analyze the tumor burden *in vivo*. **(B)** Kaplan-Meier survival curves of mice bearing LLC tumors with and without Atrx deficiency after anti-PD1 or anti-CTLA4 treatment. Neither aCTLA4 (n = 4) nor aPD1 (n = 4) treated mice showed a significant survival difference in Atrx-expression mice, compared with control group (n = 4) (*P* = 0.9341, 0.9412). Both aCTLA4 (n = 4) and aPD1 (n = 4) treated mice showed a significant survival difference in Atrx-deficient mice, compared with control group (n = 4) (*P* = 0.006, 0.003). **(C)** The luciferase signals detected by IVIS in mice bearing LLC generated tumors with and without Atrx deficiency after ICI or isotype antibody treatment. Neither aCTLA4 (n = 4) nor aPD1 (n = 4) treated mice showed a significant signal difference in Atrx-expression mice, compared with control group (n = 4) (*P* = 0.8521, 0.7644). Both aCTLA4 (n = 4) and aPD1 (n = 4) treated mice showed a significant signal difference in Atrx-deficient mice, compared with control group (n = 4) (*P* = 0.005, 0.002). **P<0.01. n.s., not significant.

To test whether loss of Atrx sensitized tumors to ICIs, we generated mice models bearing Lewis lung cancer (LLC) tumors that lacked Atrx (LLC-sgAtrx) and compared their growth with control LLC tumors (LLC-sgNTC) *in vivo* (**Figures 3A** and **4A**). In the subcutaneous mouse tumor model, the LLC-sgNTC tumor grew equivalently to the control group after anti-PD1 and anti-CLTA4 intervention. However, the tumor volume of LLC-sgAtrx was significantly decreased after anti-PD1 and anti-CTLA4 intervention compared with the control group (**Figure 3B**). In the metastatic tumor model, the mice bearing LLC-sgNTC tumors had a median survival of 24 weeks, and 100% of the mice died within 35 weeks. Neither anti-PD1 nor anti-CTLA4 agent prolonged the survival of the mice. However, both anti-PD1 and anti-CTLA4 agent significantly prolonged survival in mice bearing LLC-sgAtrx tumors (**Figure 4B**). Further, we assess the tumor burden *in vivo* by monitoring the bioluminescence signals. It is found that luciferase signals showed no decrease after anti–PD-1 and anti-CTLA4 intervention in mice bearing LLC-sgNTC tumors. The luciferase signals decreased sharply after both anti-PD1 and anti-CTLA4 intervention in mice bearing LLC-sg Atrx tumors (**Figure 4C**). These data suggest that loss of Atrx sensitizes LLC cells to both anti-PD1 and anti-CTLA4 agents.

### Alpha Thalassemia/Intellectual Disability X-Linked-Mutant Lewis lung cancer Exhibit Enhanced Infiltration of T Cells in a Tumor Microenvironment

The inflammatory status of the tumor microenvironment plays an important role in the response to immunotherapy. To further elucidate the mechanism of the enhanced immunotherapy response in Atrx-deficient tumors, we compared the immune microenvironments of LLC-sgAtrx and LLC-sgNTC tumors *via* flow cytometry. We found that LLC-sgAtrx tumors had significantly increased CD45+ immune cell and CD3+ T cell infiltration in the microenvironment after both anti-PD1 and anti-CTLA4 intervention compared with the control group (P < 0.01; **Figure 3C**). However, the infiltration of immune cells in LLC-sgNTC tumors was not significantly elevated (**Figure 3C**). These data demonstrated that *Atrx* deficiency improved immune-cell infiltration after ICI intervention.

### Alpha Thalassemia/Intellectual Disability X-Linked Deficiency Leads to Elevated IFN-γ-Stimulated Antigen Presentation and T Cell Killing

**Figure 5:**
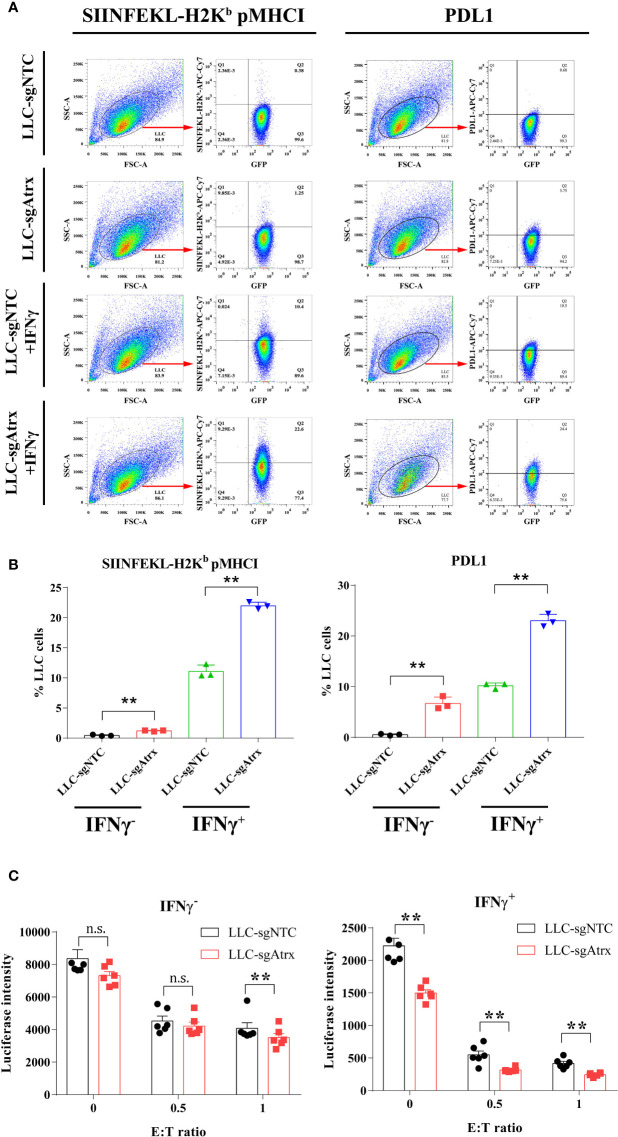
Effect of Atrx deficiency on antigen presentation and T cell cytotoxicity. **(A)** Representative flow cytometry analysis of peptide-MHC I and PDL1 surface expression under IFNγ treatment in LLC cell lines with and without Atrx deficiency. **(B)** Statistic analysis of the flow cytometry data of the expression of peptide-MHC I and PDL1 on the surface of LLC cells. Without IFNγ treatment, pMHC I expression was significantly elevated in LLC-sgAtrx cells, compared with LLC-sgNTC cells (n = 8, P = 0.007). Moreover, after IFNγ treatment, pMHC I expression was more significantly elevated in LLC-sgAtrx cells, compared with LLC-sgNTC cells (n = 8, P = 0.002). **(C)** T cell cytotoxicity assay by coculturing cognate effective T cells with Atrx mutant or non-mutant LLC cells, treated with 0 or 10 ng/ml IFNγ. Without IFNγ treatment, the T cells cytotoxicity showed no significant difference in LLC-sgNTC and LLC-sgAtrx cells at a E:T ratio of 0 (n = 5, P = 0.7012), and 0.5 (n = 6, 0.8529), while the T cell toxicity was significantly higher in LLC-sgAtrx cells, compared with LLC-sgNTC cells, at an E:T ratio of 1 (n = 6, P = 0.007). Under IFNγ treatment, the T cells cytotoxicity was significant higher in LLC-sgAtrx cells, compared with LLC-sgNTC cells at a E:T ratio of 0 (n = 5, P = 0.0081), 0.5 (n = 6, P = 0.0066), and 1 (n = 6, P = 0.0073). **P<0.01. n.s., not significant.

Loss of Atrx elevated the expression levels of pMHCI and PDL1 in LLC cells, and the elevation was further expanded under IFNγ treatment (**Figures 5A, B**), which demonstrated that the antigen-presenting ability is enhanced after Atrx deficiency. Lymphocyte coculture experiments further found that LLC-sgNTC cells showed no significant growth inhibition after IFNγ treatment and only showed significant T cell killing activity atan effector:target (E:T) ratio of 1 (**Figure 5C**). However, LLC-sgAtrx cells were significantly inhibited by IFNγ and showed significant T cell killing activity at an E:T ratio of 0.5 (**Figure 5C**). These data demonstrated that Atrx deficiency enhanced T cell killing of LLC tumors and increased its sensitivity to cytokines such as IFNγ.

## Discussion

In the present study, we demonstrated that loss of Atrx, a tumor suppressor gene that is recurrently mutated across multiple human cancers, potentiates response to ICI therapies in NSCLC. The underlying mechanisms were elucidated, including the increased T cell infiltration in the tumor microenvironment, the elevated IFN-γ-stimulated antigen presentation on tumor cells and increased killing efficacy by CD8 T cells. Although ICIs have achieved significant success in the treatment of various cancers, the effectiveness of single agents remains low. Therefore, identifying biomarkers to further stratify patients and exploring combined treatment strategies are the major challenges for clinicians. The SWI/SNF family is a group of proteins involved in chromatin accessibility regulation and plays an essential role in multiple cellular processes. With the development of genome sequencing technology, SWI/SNF subunit mutation has been revealed to occur at high frequency, in nearly 25% of all cancers (11). There are at least nine genes encoding SWI/SNF subunits that have been found to be mutated in various cancers, including SMARCB1 (12), ARID1A (13, 14), and PBRM1 (15). Atrx is another SWI/SNF family gene that is prevalently mutated in glioma (16) and soft tissue sarcomas (17). Recently, the role of SWI/SNF gene mutation in ICI sensitivity has been reported. In a clinical trial of renal cell carcinoma patients treated with anti-PD1 or anti-PD-L1 alone, or in combination with anti-CTLA4, PBRM1 mutation is related with a higher ORR and longer survival (18). Preclinical data also demonstrated that ARID1A mutation sensitizes anti-PDL1 in several types of cancer (19). In the present study, we first reported that mutation in Atrx sensitizes lung cancer to both anti-PD1 and anti-CTLA4, which indicates that Atrx may act as a biomarker of immune therapy and facilitate patient stratification.

The underlying mechanism of SWI/SNF gene mutation in improving the effectiveness of ICI is complex. It is reported that ARID1A mutant tumors had increased levels of PD-L1 expression and higher levels of cytotoxic T cell infiltration in the microenvironment (19). Loss of ARID2 or BRD7 enhanced cytokine secretion in response to IFNγ and sensitized melanoma cells to T cell-mediated cytotoxicity *in vitro* (20). In the present study, we demonstrated that Atrx mutation facilitated T cell infiltration into the LLC tumor microenvironment, elevated PD-L1 and pMHC I expression on LLC cell surfaces, and enhanced T cell cytotoxicity *in vitro*, which is in accordance with previous reports in other SWI/SNF genes. Moreover, Atrx mutation is reported to promote alternative lengthening of telomeres (ALT) in glioma (21) and is linked to DNA damage and replicative stress (22). Therefore, the mechanism of Atrx mutation sensitizing lung cancer to ICI is worth further investigation.

The prognostic role of Atrx mutation in glioma patients has been reported (8). In a recent retrospective study, Atrx was also reported to be correlated with the prognosis of lung cancer patients, including squamous cell carcinoma, adenocarcinoma and small-cell lung cancer (23). The major treatment modalities of the patients were operation, with or without adjuvant chemotherapy and/or radiotherapy, and immune therapy was not included. In the present study, we found that ATRX could sensitize mice LLC models to ICIs. Therefore, it indicated that the immune system in NSCLC patient with ATRX mutation maybe more active against cancer cells, which prolonged the control of tumor and bring benefits to the survival of patient. However, in our study, Atrx mutation is not related to patient prognosis according to TCGA data. The discrepancy may be due to the bias of stage and treatment regime in TCGA database. Hence, the prognostic role of Atrx in lung cancer patients still needs further investigation.

In summary, the present study demonstrated that Atrx mutation sensitizes LLC to ICIs. Our study reveals a potential biomarker of ICI treatment and could help with patient stratification and therapeutic decisions.

## Conclusion

The present study demonstrated that Atrx deficiency sensitize lung cancer cells to ICIs by multiple mechanisms. And ATRX may serve as a promising biomarker for ICIs. Patients with ATRX mutation may benefit more from immunotherapy, and ATRX gene analyzing may need to be incorporated into the testing panel to further facilitate patient stratification and decision making.

## Data Availability Statement

The raw data supporting the conclusions of this article will be made available by the authors, without undue reservation.

## Author Contributions

TH and SJ conceptualized and designed the study. JM, XL, and CH provided administrative support. HZ, TF, FM, and JM provided the study materials or patients. SJ, YW, YX, and XL collected and assembled the data. TH, SJ, YW, YX, and CH analyzed and interpreted the data. All authors wrote the manuscript. All authors contributed to the article and approved the submitted version.

## Conflict of Interest

The authors declare that the research was conducted in the absence of any commercial or financial relationships that could be construed as a potential conflict of interest.
