# Corrigendum: Alpha Thalassemia/Intellectual Disability X-Linked Deficiency Sensitizes Non-Small Cell Lung Cancer to Immune Checkpoint Inhibitors

**DOI:** 10.3389/fonc.2021.705406

**Published:** 2021-06-02

**Authors:** Tao Hou, Shun Jiang, Yapeng Wang, Yangchun Xie, Haixia Zhang, Yeqian Feng, Fang Ma, Jin’an Ma, Xianling Liu, Chunhong Hu

**Affiliations:** Department of Oncology, The Second Xiangya Hospital, Central South University, Changsha, China

**Keywords:** lung cancer, immune checkpoint inhibitor, CRISPR, tumor suppressor gene, α-thalassemia/intellectual disability syndrome x-linked

In the original article, there was a mistake in **Figure 4** as published. The first image in the “anti-PD-1” row was incorrect. The corrected **Figure 4** appears below.

**Figure 4:**
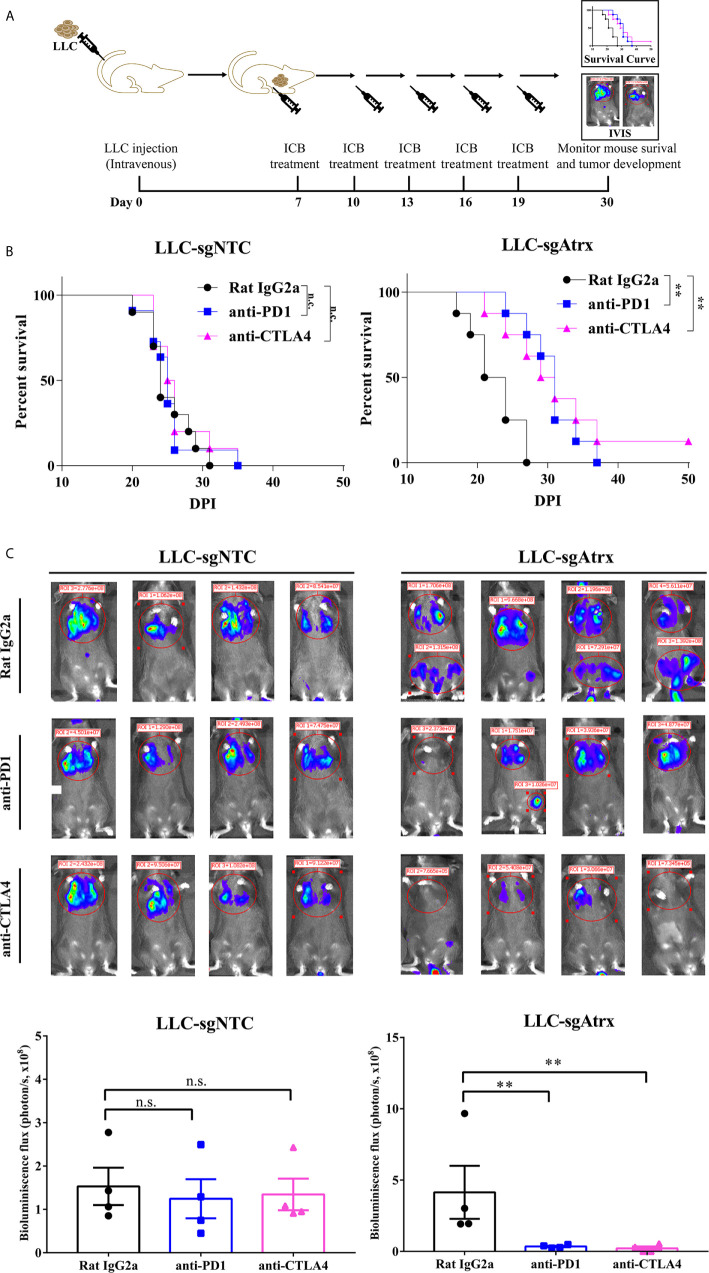
Atrx deficiency sensitizes NSCLC to ICI treatment in orthotopic mouse model. **(A)** experimental design for establishment of the orthotopic mouse model by intravenous seeding of tumor cells to analyze the tumor burden *in vivo*. **(B)** Kaplan-Meier survival curves of mice bearing LLC tumors with and without Atrx deficiency after anti-PD1 or anti-CTLA4 treatment. Neither aCTLA4 (n = 4) nor aPD1 (n = 4) treated mice showed a significant survival difference in Atrx-expression mice, compared with control group (n = 4) (P = 0.9341, 0.9412). Both aCTLA4 (n = 4) and aPD1 (n = 4) treated mice showed a significant survival difference in Atrx-deficient mice, compared with control group (n = 4) (P = 0.006, 0.003). **(C)** The luciferase signals detected by IVIS in mice bearing LLC generated tumors with and without Atrx deficiency after ICI or isotype antibody treatment. Neither aCTLA4 (n = 4) nor aPD1 (n = 4) treated mice showed a significant signal difference in Atrx-expression mice, compared with control group (n = 4) (P = 0.8521, 0.7644). Both aCTLA4 (n = 4) and aPD1 (n = 4) treated mice showed a significant signal difference in Atrx-deficient mice, compared with control group (n = 4) (P = 0.005, 0.002). **P < 0.01. n.s., not significant.

The authors apologize for this error and state that this does not change the scientific conclusions of the article in any way. The original article has been updated.

